# Bioengineering of novel organotypic 3D human liver tissue model for drug-induced liver injury and toxicity studies

**DOI:** 10.3389/ftox.2025.1574387

**Published:** 2025-06-12

**Authors:** Camden Holm, Joseph Finelli, Mateo Frare, Alex Armento, Seyoum Ayehunie

**Affiliations:** MatTek Corporation, Ashland, MA, United States

**Keywords:** liver tissue model, hepatotoxicity, drug metabolism, DILI, liver spheroids liver tissue model, liver spheroids

## Abstract

**Background:**

In drug development, liver failure is the cause of approximately 30% of post marketing withdrawals of pharmaceuticals. Drug-induced liver injury (DILI) remains the leading cause of acute liver failure (ALF), accounting for approximately 15% of the cases.

**Materials and methods:**

In this study, we developed a novel human three-dimensional (3D) liver tissue model by seeding adult primary human hepatocytes onto cell culture inserts under Air-Liquid Interface (ALI) condition for extended culture periods. The engineered tissues were thoroughly characterized for barrier integrity using transepithelial electrical resistance (TEER) measurements and assessed for tissue morphology and structure via hematoxylin and eosin (H&E) staining and immunohistochemistry. Expression levels of drug transporters and drug-metabolizing enzymes were evaluated by quantitative PCR (qPCR). The functionality of the tissue model for drug toxicity assessment was demonstrated by comparison with conventional two-dimensional (2D) monolayer hepatocyte cultures and liver spheroids. To evaluate the model's relevance for DILI studies, we exposed the 3D liver tissues to compounds with well-documented hepatotoxic profiles in humans. Liver function was monitored by quantifying biomarkers such as alanine aminotransferase (ALT) and aspartate aminotransferase (AST) released into the culture medium.

**Results:**

The engineered 3D liver tissue model exhibited distinct apical and basolateral surfaces, reflecting a polarized and stratified architecture that closely mimics native liver tissue. Morphological and phenotypic analyses confirmed the tissue's organotypic features. Gene expression profiling revealed elevated levels of liver-specific genes involved in drug transport, metabolism, and clearance. Functionally, the tissue metabolized midazolam--a substrate of the cytochrome P450 3A4 (CYP3A4) enzyme--into its primary metabolite, 1-hydroxymidazolam. Upon repeated exposure to fialuridine, a discontinued anti-hepatitis B drug known for causing severe liver toxicity in humans, the tissue model exhibited barrier compromise, reduced albumin production, and increased levels of ALT and AST in a time- and concentration-dependent manner.

**Discussion:**

The results strongly suggest the model's physiological relevance and functionality in predicting drug responses in humans. Thus, the engineered 3D organotypic human liver tissue model which can be cultured for weeks and produced in a semi-high throughput format creates an opportunity to study drug-induced liver toxicity in an in vitro microenvironment. The reconstructed 3D liver tissue model can serve as a tool for alternative methods intended to reduce animal use in experimentation.

## Graphical Abstract

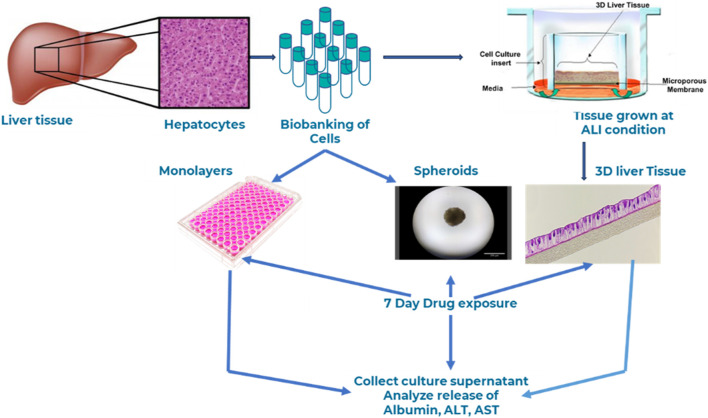

## 1 Introduction

The traditional method for drug development is an expensive and a time-consuming process that costs an estimated 2-3 billion dollars per drug over the course of 10–12 years (Horiuchi et al., 2022). The preclinical phase, which includes animal studies to evaluate safety and adverse outcomes, takes about 5–6 years. Furthermore, drugs that pass animal experimentation as safe still risk failure during clinical trial or post-market withdrawal affecting up to 30% of the cases (Tagle, 2019). Since most pharmacological agents are lipophilic and are metabolized by the liver, the liver is a prime target for drug toxicity either by the parent drug or its bioactive metabolites (Weber and Gerbes, 2022). As a result, drug-induced liver injury (DILI) remains the leading cause of acute liver failure (ALF), accounting for approximately 15% of the cases (Aithal et al., 2011; Katarey and Verma, 2016). Based on FDA-approved drug labeling and publicly available data, 18% (192 out of 1,036) of the drugs in the DILIrank dataset fall into the “Most-DILI-concern” category (Chen et al., 2016). DILI can manifest as acute or chronic, and either as hepatitis, cholestasis, or a mix of both (David and Hamilton, 2010; Reuben et al., 2010; Weber and Gerbes, 2022). DILI is currently clinically characterized by increased level of liver enzymes such as alanine aminotransferase (ALT) and aspartate aminotransferase (AST) in serum (Aithal et al., 2011; Katarey and Verma, 2016). Thus, AST) and ALT are routinely used as liver function tests to monitor drug-induced liver injury (DILI) or hepatotoxicity in patients (Weber and Gerbes, 2022. During drug development, extensive testing is performed in animals to catalog the safety and efficacy of drug candidates (Katarey and Verma, 2016). However, animal experiments do not necessarily predict human outcomes due to species differences related to drug metabolism, transport, and clearance profiles that can alter the pharmacokinetics of a drugs in humans. For instance, evidence based estimates indicate that in 2015, approximately 79.9 million experiments were conducted worldwide, involving an estimated 197 million animals (Taylor, 2024; Taylor, 2024) which raises ethical concerns and the need for alternative methods that reduces animal use for research. Despite the use of a large number of animals every year in preclinical testing, species differences exist, and many drugs that were deemed safe during animal studies later caused toxicity, including severe hepatotoxicity, in humans. For example, the anti-hepatitis B drug Fialuridine, developed in the 1990s and extensively tested in five animal models (mice, rats, dogs, monkeys, and woodchucks), failed during the 9-week clinical trials due to its unexpected induction of liver failure in humans. The trial was terminated following the deaths of five volunteers (McKenzie et al., 1995; Reuben et al., 2010; McKenzie et al., 1995; Reuben et al., 2010). Additional drugs recalled from the market or stopped during human clinical trials include iproniazid, ticrynafen, benoxaprofen, bromfenac, troglitazone, nefazodone, isoniazid, labetalol, trovafloxacin, tolcapone, felbamate ibufenac, perhexiline, alpidem, dilevalol, tasosartan, and ximelagatran (U.S. Food and Drug Administration, 2009; Babai et al., 2021).

Over the years, different cell-based assay methods have been developed to reduce animal testing and enhance the prediction of drug-induced liver toxicity. A range of *in vitro* end point measurements such as cell death, lactate dehydrogenase (LDH) release assays have been used as markers of DILI in two-dimensional cell line-based culture models. Immortalized cell lines, such as HepaRG that are widely used in hepatoxicity research (Godoy et al., 2013). *In vitro* liver models, such as liver spheroids, hepatocyte co-culture with non-parenchymal cells and other relevant cell types grown in ultra-low binding plates and microfluidic chip systems were also used in specific context-of use (Yang et al., 2023) inhepatotoxicity studies. The 3D organotypic liver tissue models have physiological and functional similarity to the *in vivo* counterpart compared to 2D monolayer cultures (Donato et al., 2022). However, the use of primary human hepatocytes (PHH) to determine DILI is hampered by: 1) the short lifespan of PHH cells in culture and 2) rapid dedifferentiation and loss of functionality of PHHs in culture, which can lead to a decrease in detoxification and clearance capability of drug compounds and albumin release, a major protein produced by the liver (Godoy et al., 2013).

Our effort initially focused on optimizing media composition and developing culture methods to increase the lifespan of PHH cells in the 3D organotypic liver cultures. Then, attention shifted to generating well differentiated and stratified three-dimensional tissues using PHHs and formulation of specific medium that can support tissue growth for over 3 weeks. The bioengineered 3D organotypic liver tissue model described in this manuscript is physiologically and functionally relevant for predicting DILI in humans. Optimizing *in vitro* culture conditions to sustain hepatocytes for longer periods without phenotypic changes also facilitated the monitoring of tissue production reproducibility and the generation of predictive datasets that reflect drug exposure responses (acute or sub-chronic), closely mimicking those of the *in vivo* counterpart.

In this study, we present the bioengineering of a novel three-dimensional (3D) human liver tissue model on Transwell inserts using primary human hepatocytes (PHHs) cultured under air–liquid interface conditions. The resulting 3D tissue is well-stratified and differentiated, exhibiting clearly defined apical and basolateral orientations. This physiologically relevant and functional model can be maintained for an extended duration up to 23–30 days compared to conventional monolayer hepatotoxicity assays, which typically last only 2 h to 5 days. Notably, the model accurately predicted the acute and sub-chronic hepatotoxic effects of Fialuridine, a test drug that appeared safe in animal studies but led to liver failure in human clinical trials. These findings suggest that this 3D liver tissue model holds significant promise as a preclinical tool for predicting drug transport, metabolism, and hepatotoxicity.

## 2 Materials and methods

### 2.1 Cell source

Human Primary Liver hepatocytes (PHHs) were purchased from Discovery Life Sciences (DLS, Woburn, Massachusetts). Cells were ethically harvested from liver tissue donated for research.

### 2.2 Test drugs

All test drugs were purchased from Sigma-Aldrich. Stock solutions of Bosentan Hydrate, Diclofenac Sodium Salt, Fialuridine, and Tolcapone were prepared in DMSO at 20 mM. Stock solutions of SN38 were prepared in DMSO at 50 mM stock concentration. In all dosing solutions, the final DMSO concentration in media was kept under 0.1%. Sources and catalog numbers of the drugs are listed in Table 1. The drugs represent a diverse range of physicochemical properties. To compare drug induced liver injury, the 3D liver tissues, spheroids, or hepatocyte monolayers obtained from the autologous donor were used to test drugs at various concentrations.

**TABLE 1:** List of drugs used in the study.

Drug Name	Source	Catalog #	Stock concentration
Bosentan Hydrate	Sigma	SML1265	20 mM in DMSO
Diclofenac Sodium Salt	Sigma	D6899	20 mM in DMSO
Fialuridine	Sigma	SML0632	20 mM in DMSO
Tolcapone	Sigma	SML0150	20 mM in DMSO
SN38	Sigma	H0165	50 mM in DMSO

### 2.3 Monolayer

PHH monolayers were seeded in 24-well plates following manufacturer protocol. Briefly, frozen cells were thawed by warming vials in a 37°C water bath for 2 minutes and the thawed cells were centrifuged at 100 g for 10 min. Cell pellets were then resuspended in media at 0.7 million cells per mL and 500 µL of the cell suspension (3.5 × 10^5^ cells)/well) was added to 24-well inserts. Hepatocytes were allowed to attach overnight in an incubator (37°C, 5% CO_2_) and then the cells were fed with a specially formulated hepatocyte differentiation medium (MatTek Life Sciences, Ashland, Massachusetts) for 14 days before dosing.

### 2.4 Spheroids

To test the capability of the donor cells to make liver spheroids, cells were thawed and seeded on a BIOFLOAT 96-well Ultra-low attachment plate (faCellitate Cat# F202003) at a density of 5,000 cells per well. Then cells were fed with a specially formulated hepatocyte culture medium (MatTek Life Sciences, Massachusetts) and left undisturbed in an incubator (37°C, 5% CO_2_) for 4 days. After day 4 of the culture period the cells aggregate to form spheroids. Spheroid images were taken every other day to monitor the size and shape of spheroids. The cells were cultured for a total of 14 days prior to the initiation of drug toxicity experiments.

### 2.5 Bioengineering of 3D liver tissue model

To reconstruct the 3D liver tissue model, cryopreserved primary human liver hepatocytes were thawed, centrifuged, and seeded (2.0 × 10^5^ cells/cm^2^) onto human collagen coated 24-well tissue culture inserts (MatTek Corporation, 0.6 cm^2^) and cultured for 3 days under submerged condition. After 3 days, tissue culture inserts were air lifted and cultured at air-liquid interface (ALI) condition in specially formulated tissue differentiation medium (LIV-100-MM, MatTek Corporation, Ashland, MA) for up to 28 days at 37°C, 5% CO2 and 98% relative humidity. During the ALI culture period, tissues were fed basolaterally with 5 mL of medium. To maintain apical surface hydration, 100 µL of the culture media was added topically on each feeding cycle.

### 2.6 Histology

To evaluate tissue structure, representative tissue reconstructs were fixed with 10% formalin overnight. The tissues were embedded in paraffin and cross-sections were made (5–7 µm thick) and stained with H&E at MatTek. H&E-stained tissues were photographed using an Olympus VS 120 scanning microscope. H&E staining was performed at various timepoints during the culture period to monitor structural features.

### 2.7 Barrier integrity measurement

To monitor tissue barrier integrity changes in barrier function of the liver tissue model were quantified using transepithelial electrical resistance (TEER) measurements as previously described (Ayehunie et al., 2018) using the EVOM volt-ohmmeter equipped with an EndOhm electrode chamber (World Precision Instruments, Sarasota, FL). TEER monitors the presence of functional intercellular tight junctions which are responsible for the barrier function in tissues. Raw resistance values (Ω) were converted to TEER readings (Ω*cm^2^) by multiplying the raw values for each tissue by the surface area of the cell culture inserts (0.6 cm^2^ for 24-well inserts or 0.12 cm^2^ for the 96-well membrane inserts). For drug treated tissues, TEER values were normalized as a percentage of the untreated control tissues: %TEER = TEER (Ω*cm2) of treated tissues (TTT) divided by the TEER of untreated tissues (TUT) times 100 (% TEER = (TTT/TUT)*100).

### 2.8 Immunostaining of 3D organotypic tissue model

To characterize expression of specific markers, immunohistochemical assays were performed using antibodies for albumin, CK7, CD-10, and ZO-1. Tissues were fixed at various timepoints of the culture period using 10% formalin for 24 h. After 24h fixation, tissues were transferred to DPBS and processed at MatTek’s histology facility. Briefly, tissues were embedded in paraffin then sectioned, and three slices of each tissue were placed on a slide and deparaffinized. For antigen retrieval, slides were rehydrated in 1x TBS then incubated in citraconic anhydride (Alfa Aesar Cat# L05238) pH7.4 at 98°C for 45 min. Slides were then allowed to cool at room temperature in the citrate buffer for an additional 20 min. The slides were washed in 1x TBS, then blocked in 10% goat serum with 1% BSA in TBS for 1 h. Samples were incubated with primary antibody (Mouse anti-Human Albumin Monoclonal antibody: Abcam Cat# ab236492; Rabbit anti- Human ZO-1 Polyclonal antibody: Invitrogen Cat# 61–7,300; Mouse anti-Human CD10 Monoclonal antibody: Abcam Cat# ab951) at the manufacturers recommended concentration for 1 h at room temperature, then washed with TBS. Samples were incubated in secondary antibody (Goat anti-Rabbit IgG (H + L) Cross-Adsorbed Secondary Antibody, Alexa Fluor™ 488: Invitrogen; Cat# A11008; Goat anti-Mouse IgG (H + L) Cross-Adsorbed Secondary Antibody, Alexa Fluor™ 555: Invitrogen; Cat# A21422) at the manufacturers recommended concentration for 1 h at room temperature in the dark, then washed with TBS. Samples were rinsed with deionized sterile water, then coverslips were mounted using ProLong Gold antifade mounting medium containing DAPI (Invitrogen Cat# P36931). Samples were allowed to cure overnight and were imaged using a Revolve ECHO microscope (Discover Echo, San Diego).

### 2.9 Ribonucleic acid (RNA) isolation and quantification

Tissues (N = 2) were rinsed three times with phosphate buffered saline and, together with the underlying membrane, cut from the 24-well plate using a scalpel, and deposited into a PCR clean microcentrifuge tube with lysis/binding buffer (RNeasy Plus Mini Kit Cat# 74134, Qiagen, Germantown, MD). Tissues were homogenized using a pellet pestle and total RNA was isolated following the manufacturer recommendations. Trace DNA contamination was removed using gDNA Eliminator spin columns (Qiagen). RNA was isolated on days 9, 16, and 23 of the culture periods. RNA concentrations were quantified using spectrophotometry at 260 nm and purity was verified using the 260/280 nm ratio on a NanoDrop8000. This procedure yielded between 3 and 5 μg of high-quality total RNA per/tissue. RNA was stored at −80C until use.

### 2.10 Reverse transcription polymerase chain reaction (RT-PCR)

RT-qPCR was performed to investigate gene expression levels of drug transporters and drug metabolizing enzymes (phase I and II). Total RNA (500 ng) isolated from 3D liver tissues was reverse transcribed using the RT^2^ First Strand Kit (Qiagen Cat# 330404) following the manufacturer’s recommendations. The cDNA synthesis was performed on an Eppendorf Mastercycler Gradient (Eppendorf, Enfield CT) by incubating samples with reverse transcriptase enzyme at 42°C for 15 min, followed denaturation by incubating the cDNA at 95°C for 5 min. This protocol was chosen to minimize nonspecific product amplification. Then, 1.25 µL of the cDNA product was mixed with 13 µL of nuclease-free H20 and 14 µL RT^2^ SYBR Green qPCR Mastermix (Qiagen Cat# 330503). This volume was then added to each of 96-wells in RT^2^ Profiler™ PCR Arrays (Qiagen Cat# PAHS-068ZD-24, PAHS-069ZD-24, Cat# PAHS-070ZD-24) and an RT2 PCR program was run following manufacturer protocol on each array using a BioRad C1000 Thermal Cycler. The PCR cycle used was 1-min duration at 94°C (denaturation), 1 min at 58°C (annealing), and 1 min at 72°C (extension) for 40 cycles, Then, a final extension at 72°C for 10 min was used followed by a hold temperature at 4°C. Glyceraldehyde-3-phosphate dehydrogenase (GAPDH) was used as the amplification internal control. Genes with a Cq value below 30 cycles were considered to be highly expressed by the tissue model.

### 2.11 Quality control

To monitor the reproducibility of each lot of 3D liver reconstructs for the various experiments, quality assurance specifications were established. TEER was used as an end point measurement to ensure lot-to-lot reproducibility. TEER values of >70 Ω*cm^2^ were set as an acceptance criteria for each lot of the liver tissue reconstruct to be used for the studies.

### 2.12 Drug metabolism

To evaluate drug metabolism by the reconstructed tissue model, the 3D liver tissue was dosed apically with the test drug Midazolam (100 µM) for 2 hours. After 2 h, 200 µL culture media was added to apical surface and both the apical washes and basolateral supernatants were collected and stored at −80°C for mass spectrometry analysis using Liquid chromatography–tandem mass spectrometry (LC/MS/MS). Mass spectrometry analysis was performed for both apical washes and culture supernatants. Supernatants were analyzed for the presence of a Midazolam metabolite, 1-OH-midazolam. Detection of the metabolite in the apical washes or in the culture supernatant indicates functional CYP3A activity.

### 2.13 Comparison of *in vitro* liver models for DILI or toxicity

To compare drug induced liver injury of the reconstructed 3D liver tissue model with that of monolayers and liver spheroids, we dosed monolayer cells, spheroids, and the 3D organotypic liver tissue (cells obtained from the autologous donor) with two model drugs using N = 2 tissues/drug/concentration. Monolayers were dosed with 500 µL of media containing drugs; spheroids were dosed with 100 µL of media containing the drugs, and 3D reconstructed tissues were dosed apically with 100 µL media containing drugs at days 0, 2, and 4. Apical washes and culture supernatants were collected on days 2, 4, and 7 post drug exposure. Outcome measurements for liver injury or toxicity include releases of albumin, ALT, and AST as measured by specific ELISA.

### 2.14 DILI modeling using engineered 3D liver tissue model

Drug induced liver toxicity was further demonstrated by dosing the 3D organotypic liver tissue model with expanded list of test drugs that includes SN38 (20 µM), Bosentan (100 µM), Diclofenac (50 µM), Fialuridine (100 µM), and Tolcapone (100 µM). The 3D reconstructed tissues (N-2 tissues per drug/concentration were exposed to drugs apically (100 µL) and basolaterally (5 mL). Then, supernatants were collected on days 2 and 7 post drug exposure. Outcome measurements for liver toxicity include levels of albumin, ALT, and AST release in apical washes and/or to culture supernatants.

### 2.15 Enzyme-linked immunosorbent assay (ELISA)

Tissue culture supernatants were collected at various timepoints during tissue growth and throughout dug treatment period. Supernatants were stored at −20°C until ELISA assays were performed. The presence of albumin was quantified by a human albumin ELISA from Invitrogen (Human Albumin ELISA Kit Cat# EHALB). The presence of AST, and ALT was quantified by human AST and ALT ELISAs from Abcam (Human AST ELISA Kit Cat# ab263881, Human ALT ELISA Kit Cat# ab234578) following the manufacturers’ recommendation. Supernatants were diluted 1:1,000 for albumin and 1:2 for AST and ALT ELISA assays. For each test, samples were run in duplicate.

## 3 Results

### 3.1 Tissue structure and characterization

**FIGURE 1:**
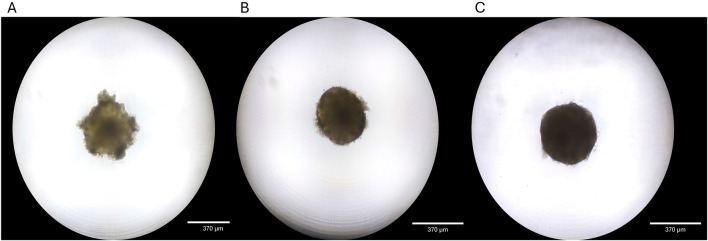
Liver spheroid development. **(A)** Spheroid 4 days after seeding. **(B)** Spheroid is fully formed at 7 days in culture. **(C)** Spheroids maintain structure and grow for 14 days in culture.

**FIGURE 2:**
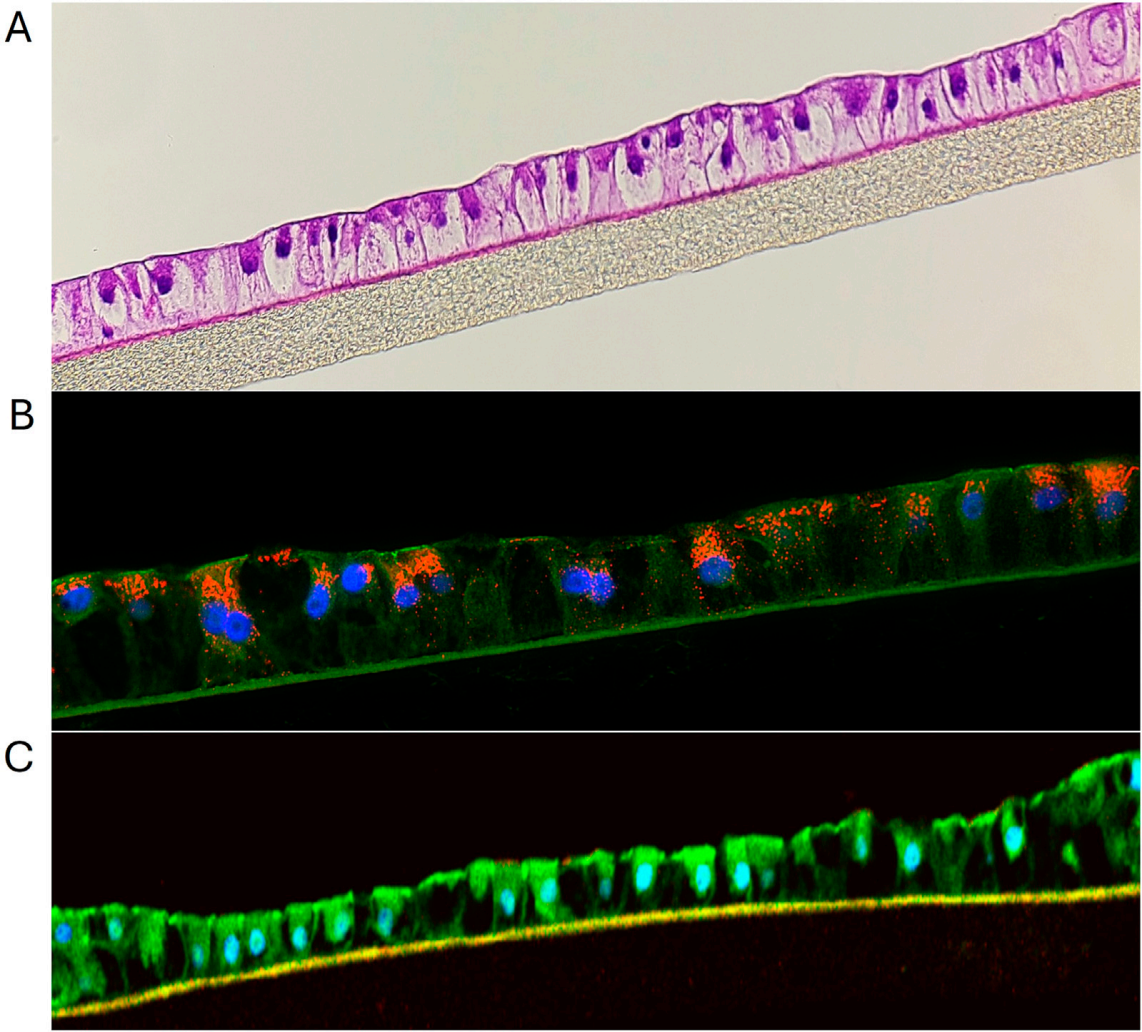
**(A)** H&E staining showing structural features of liver tissue model. **(B)** Immunohistochemical staining of liver tissues at 10 days in culture. The red stain is albumin, green stain is Z0-1, blue stain is nuclei with DAPI. **(C)** IHC showing ZO-1 expression (green) by liver tissue model.

**FIGURE 3:**
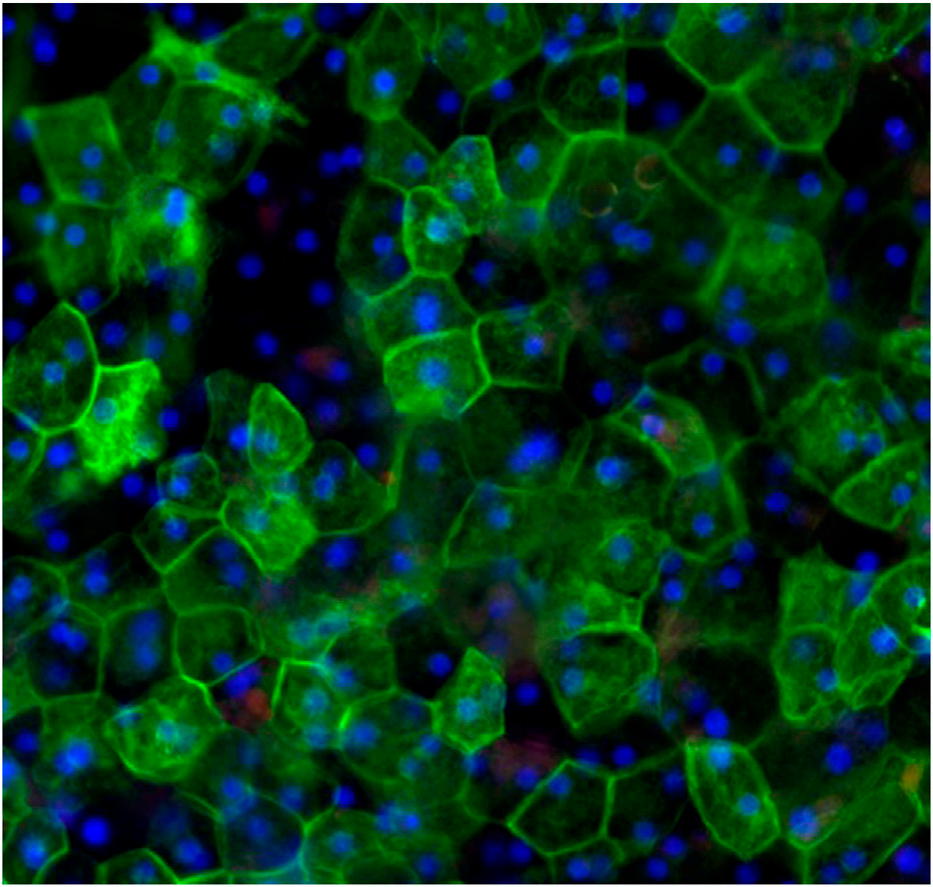
Topical view of immunocytochemical staining of liver tissues at 20 days in culture demonstrating hexagonal cellular structure and organization of hepatocytes. Red stained albumin, green stained epithelial cell marker (CK-7), blue stained nuclei, stained with DAPI.

**FIGURE 4:**
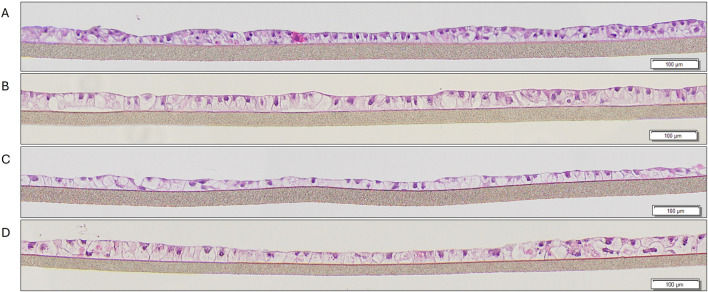
H&E staining of liver tissues at various timepoints in culture: **(A)** 9 days, **(B)** 10 days, **(C)** 14 days, **(D)** 21 days.

Cryopreserved primary human liver hepatocyte monolayers were used to generate liver spheroids (Figure 1) or 3D organotypic human liver tissue models. For the 3D liver model, hepatocytes were seeded into single-wells of 24-well tissue culture inserts for 3 days under submerged condition and then cultured at ALI condition to produce 3D organotypic liver tissue model. Histological and fluorescent microscopy imaging (Figures 2–4) of the reconstructed 3D liver tissues showed in vivo-like architecture consisting of well polarized and differentiated columnar hepatocyte layer that is positive for cytokeratin-7 (bile duct epithelial cell and hepatocyte progenitor marker).

### 3.2 Transepithelial electrical resistance (TEER)

In this study, TEER is used to assess barrier integrity in the 3D liver tissue model, and the mean TEER values across four experiments were 194.93 ± 70.5 Ω*cm2 at day 14 of the culture period and 251.33 ± 114.99 Ω*cm2 at day 21 (Table 2). The range of TEER values obtained is slightly lower than the TEER value reported for HepG2 monolayer cells (TEER value = 345–395 Ω*mm^2^ (Farooqi et al., 2021).

**TABLE 2:** TEER measurements across four experiments.

Experiment	Days of culture					
	14			21		
	TEER	Mean TEER	STD	TEER	Mean TEER	STD
L22-1	187.5	194.93	70.50	408.3	251.33	114.99
L23-2	207.9			258.3		
L25-3	106.5			141.6		
L29-4	277.8			197.1		

To monitor the suitability of each preparation of 3D liver reconstructs, quality assurance specifications were established using TEER measurements. A TEER value of >70 Ω*cm2 was set as an acceptance criterion for each lot of tissue production.

### 3.3 Immunostaining of 3D organotypic tissue model

**FIGURE 5:**
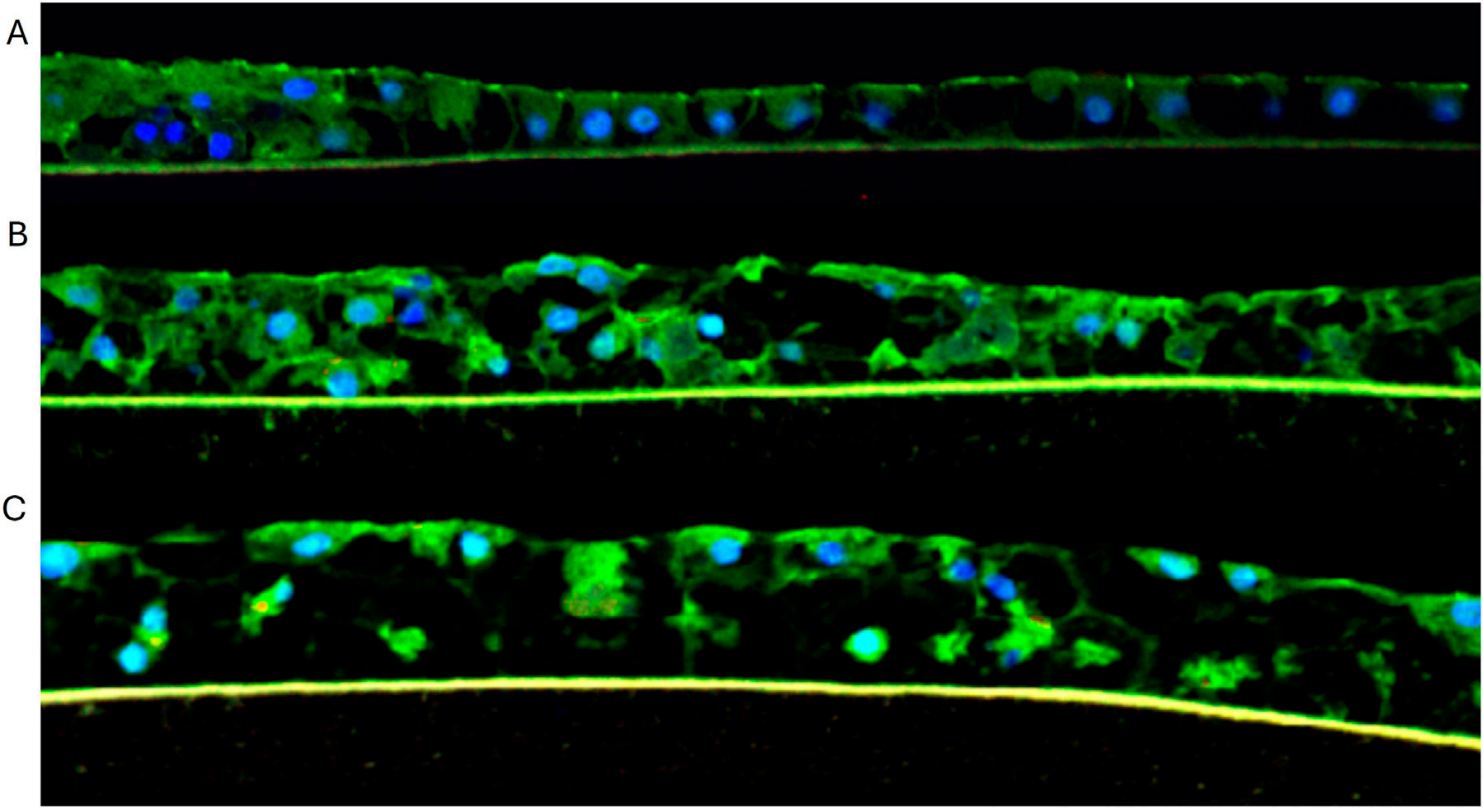
Immunocytochemical staining of liver tissues at **(A)** 10, **(B)** 16, and **(C)** 21 days in culture. Green = Z0-1 staining; Blue = nuclei stained with DAPI.

The reconstructed 3D liver tissues were characterized by staining for albumin, CK7, CD-10, and ZO-1. Nuclei were stained with DAPI. Immunostaining results showed tissue architecture with columnar alignment of PHHs. Positive albumin staining appeared on the apical side of the epithelial layer (Figure 2), further demonstrating tissue polarization. The presence of ZO-1 (tight junction marker, (Nakamura, 2013), by the reconstructed tissues indicates well developed cellular junction and apical polarity (Figure 5).

### 3.4 Gene expression of drug transporters and drug metabolizing enzymes

Drug transporters (e.g., P-gp) and drug metabolizing enzymes (e.g., CYP3A4) can influence the amount of bio-active drug and metabolites that affect liver itself or distant target organs. In this study, we investigated the key efflux transporters including ABCB1/MDR1 (P-glycoprotein, P-gp), ABCC1 (multidrug resistance protein (MRP)-1), ABCC2 (MRP-2), and ABCG2 (breast cancer multidrug resistance protein, BCRP). RT-PCR results showed that the 3D liver tissue expresses the major efflux drug transporters known to be present in human liver at different times of the culture period (Supplementary Material S1).

Since drug metabolism is one of the vital functions of the liver (e.g., CYP3A4), we investigated the presence of the main drug metabolizing enzymes in the 3D liver tissue model. RT-qPCR results showed that the tissue model expresses the Phase I drug metabolizing enzymes known to be present in in human liver (Supplementary Material S2). The Phase I drug metabolizing enzymes present in the liver and expressed by the *in vitro* liver tissue model include CYP2C8, CYP2C9, CYP2C18, CYP2C19, CYP2D6, CYP2E1, CYP3A4, CYP3A5, CYP3A7, and CYP4A1. In addition, the 3D liver tissues expressed Phase II drug detoxification enzymes such as glutathione S-transferases which are involved in the inactivation of bioactives at levels similar to the expression pattern seen in the native liver tissue (Supplementary Material S3), However, additional work is needed to verify the functionality or activity of Phase II enzymes using multiple substrates. In addition to directly affecting the amount of bioactive drug, expression of these efflux transporters and Phase II drug-metabolizing enzymes can play a role in drug clearance.

### 3.5 Drug metabolism

To examine the functionality of the 3D liver model, tissues were exposed to media or dosed with 100 µM midazolam for 2 h and supernatants and apical washes were collected for analysis. LC/MS/MS analysis of supernatant and apical washes from dosed reconstructed 3D tissues showed Midazolam was metabolized and one of its metabolites, 1-OH-midazolam (Table 3), was detected in both apical washes and culture supernatants. The results showed that the main liver drug metabolism enzyme CYP3A4 is functional in the reconstructed tissue model.

**TABLE 3:** Midazolam metabolism to its metabolite 1-hydroxymidazolam by 3D liver tissue model.

Media	Drug	Day of treatment	Sample Name	Analyte Peak area (counts)	IS Peak area (counts)	Analyte/IS	Calculated amount (nM)
Apical	Control	12	8a_AP_CT_2	2.04E+03	1.79E+06	1.14E-03	0.00
	Midazolam	12	8a_AP_MD_3	7.43E+05	1.77E+06	4.20E-01	148.86
		15	11a_AP_MD_4	5.65E+05	1.68E+06	3.36E-01	116.81
		22	18a_AP_MD_6	6.53E+05	2.19E+06	2.98E-01	102.17
Basolateral	Control	12	8a_BS_CT_7	1.26E+03	1.84E+06	6.85E-04	0.00
	Midazolam	12	8a_BS_MD_8	1.89E+05	2.46E+06	7.68E-02	257.6
		15	11a_BS_MD_10	1.67E+05	2.44E+06	6.84E-02	209.3
		22	18a_BS_MD_11	1.25E+05	1.98E+06	6.31E-02	178.7

### 3.6 Comparisons of monolayers, spheroids, and organotypic liver tissue models for drug induced liver toxicity

**FIGURE 6:**
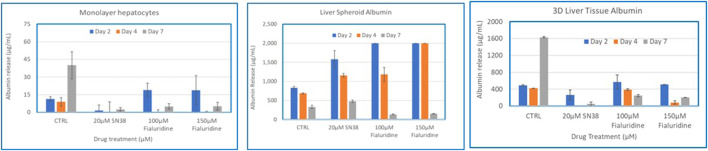
Comparison of Drug-Induced Albumin Release in Human Hepatocyte Monolayers, Liver Spheroids, and 3D Liver Tissue Models. Human hepatocyte monolayers, liver spheroids, and 3D liver tissue models were compared for drug toxicity. The 3D liver tissue models were cultured under air-liquid interface (ALI) conditions for 14 days in tissue culture inserts. Following this, the models were exposed to test drugs every other day over a 7-day period. For all model systems duplicate wells per drug/concentration (N = 2 was used. On days 2, 4, and 7 culture supernatants were collected, and albumin levels were quantified using a albumin specific ELISA kit. The results were then compared across the three model systems to assess differences in drug-induced albumin release. There was a difference among the treatment groups (p value < 0.0001; Two-way ANOVA) at day 7 post exposure.

**FIGURE 7:**
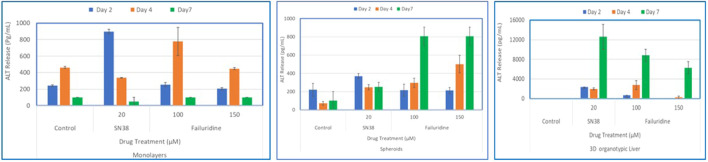
Comparison of Drug-Induced ALT Release in human hepatocyte Monolayers, liver spheroids, and 3D Liver tissue models. Human hepatocyte monolayers, liver spheroids, and 3D liver tissue models were compared for drug toxicity. The 3D liver tissue models were cultured under air-liquid interface (ALI) conditions for 14 days in tissue culture inserts. Following this, the models were exposed to test drugs every other day over a 7-day period. For all model systems duplicate wells per drug/concentration (N = 2 was used. On days 2, 4, and 7 culture supernatants were collected and released ALT levels were quantified using an ALT specific ELISA kit. The results were then compared across the three model systems to assess differences in drug-induced albumin release. There was a difference among the treatment groups (p value < 0.0001; Two-way ANOVA) at day 7 post exposure.

**FIGURE 8:**
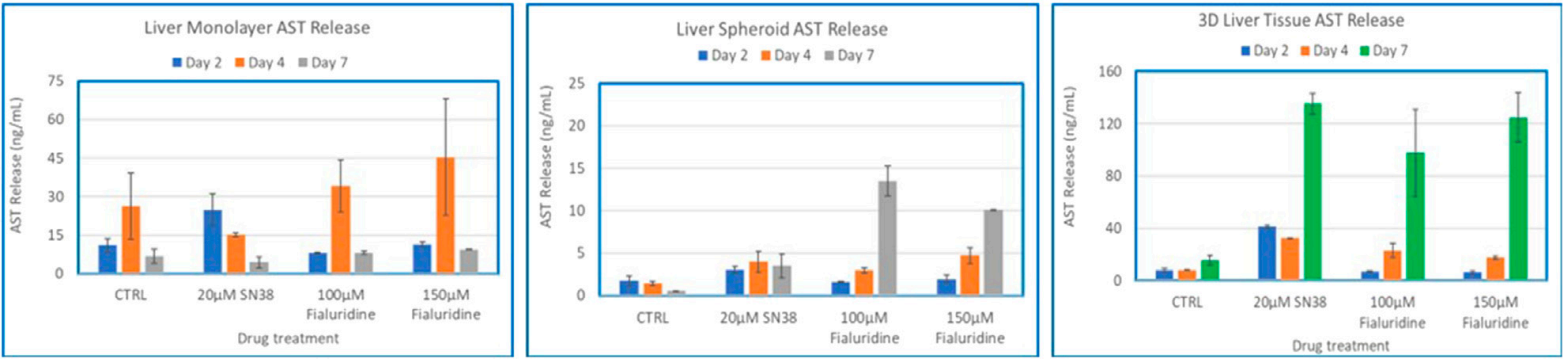
Comparison of Drug-Induced AST Release in human hepatocyte Monolayers, liver spheroids, and 3D Liver tissue models. Human hepatocyte monolayers, liver spheroids, and 3D liver tissue models were compared for drug toxicity. The 3D liver tissue models were cultured under air-liquid interface (ALI) conditions for 14 days in tissue culture inserts. Following this, the models were exposed to test drugs every other day over a 7-day period. For all model systems duplicate wells per drug/concentration (N = 2 was used. On days 2, 4, and 7 culture supernatants were collected and released ALT levels were quantified using an ALT specific ELISA kit. The results were then compared across the three model systems to assess differences in drug-induced AST release. There was no difference among the treatment groups (p value < 0.08, Two-way ANOVA) at day 7 post exposure.

To compare the 3D organotypic reconstructed tissues with that of primary hepatocyte monolayers and spheroids for drug toxicity studies, cells/tissues were dosed with SN38 (20 µM) and Fialuridine (100µM and 150 µM). The different hepatocyte models were dosed with the drugs for 7 days and then culture supernatants were collected for analysis. The result showed albumin production was very low in monolayer culture (Figure 6). The spheroids showed an increase in albumin production in response to repeated drug exposure until day 7, whereas the engineered 3D reconstructed liver tissues showed a decrease in albumin production following repeat drug exposure (Figure 6). Furthermore, culture supernatants from all tissue types treated with the drugs and untreated controls were collected and analyzed to determine levels of two clinically relevance biomarkers used in liver function tests, AST and ALT. The result showed a 3-4-fold increase in AST and ALT release by day 7 for the 3D organotypic liver tissue model for Fialuridine compared to monolayers and spheroid cultures (Figures 7, 8). This demonstrated that the effect of the clinically failed drugs like Fialuridine in humans could have been predicted prior to human clinical trials if such models were available at the time.

### 3.7 Modeling DILI using the engineered 3D liver tissue model

**FIGURE 9:**
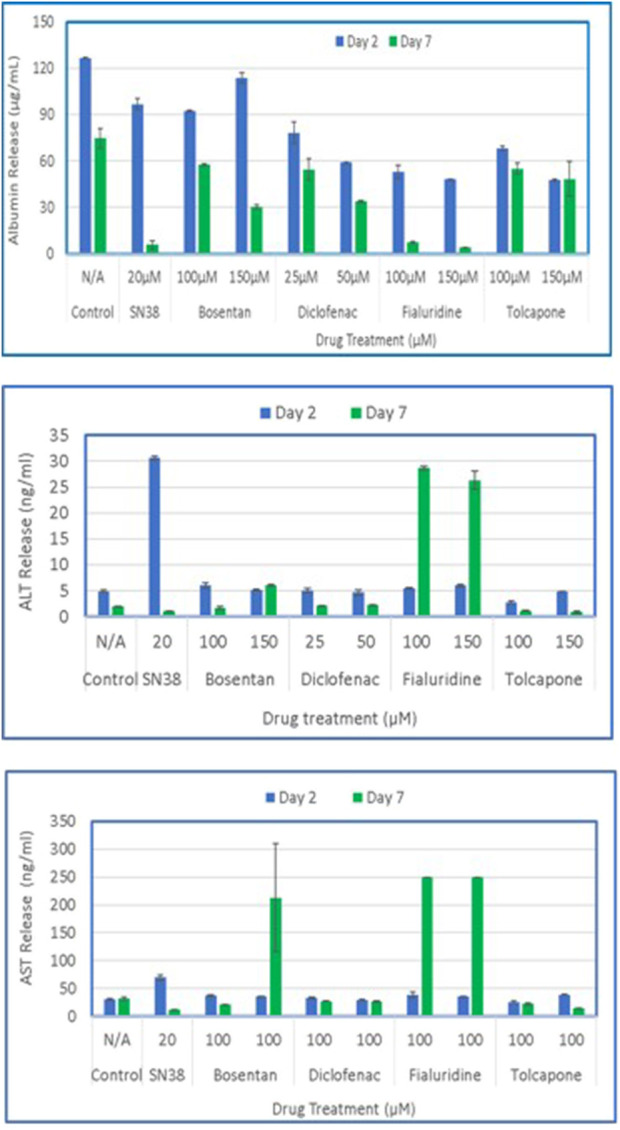
Drug induced release of Albumin, ALT and AST by the 3D liver tissue model. Tissues cultured for 14 days were dosed to test drugs every other day (N = 3 repeat doses). Culture supernatants were collected on days 2 and 7 and analyzed by specific ELISA for released levels of albumin, ALT, and AST.

To model DILI *in vitro*, the 3D organotypic reconstructed liver tissues were exposed to two concentrations of 5 model drugs: Bosentan (100µM and 150 µM), Diclofenac (25µM and 50 µM), Fialuridine (100µM and 150 µM), and Tolcapone (100µM and 150 µM). SN38 (20 µM) was included as a positive control and untreated tissues exposed to media only serves as a negative control. For analysis, apical washes from negative controls and tissues dosed with test drugs were collected by adding 200 µL of media to the apical surface. One ml of basolateral supernatant was also collected on days 2 and 7 post drug exposure. For the 7-day study, tissues were re-dosed on days 2 and 5. ELISA assays were performed to determine released levels of albumin, AST, and ALT into the culture supernatant. The result showed that tissues dosed with the positive control (SN38-treated) have slightly less albumin release compared to control tissues on day 2, and diminished on day 7, possibly due to cell death by the test agent. On day 7 post-exposure, tissues treated with Bosentan, Diclofenac, Fialuridine and Tolcapone showed less albumin secretion compared to the untreated control tissue. The positive control tissues had increased levels of AST and ALT on day 2, with lower release on day 7. Bosentan, Diclofenac, and Tolcapone treated tissues showed little change in AST/ALT release compared to negative control tissues (Figure 9). However, the liver toxicant Fialuridine-treated tissues showed a significant decrease in albumin production and an increase in levels of AST/ALT secretion at day 7 post-exposure, demonstrating the predictive potential of the tissue model for drug-induced liver toxicity studies as observed during the 9-week clinical trial of Fialuridine (Institute of Medicine Committee to Review the Fialuridine Clinical Trials, 1995).

## 4 Discussion

The purpose of this study is to engineer a human primary hepatocyte cell-based 3D liver tissue model that is physiological and functional, capable of being cultured for weeks without phenotypic changes. In addition to metabolic functionality, we measured barrier integrity using transepithelial electrical resistance (TEER) as an end point for drug induced liver toxicity. This paper describes a novel primary human hepatocyte cell-based 3D tissue model to predict DILI compared to monolayer and spheroids. The culture time of the reconstructed liver organotypic tissue model is significantly longer than primary hepatocyte monolayer cultures, making sub-chronic liver toxicity experiments possible. The reconstructed tissue retains expression of vital drug metabolizing enzymes and transporters for up to 21 days, which is better than monolayer hepatic cell cultures that typically last for less than 5 days. The easy-to-use 24-well or 96 well-format of this tissue model makes it suitable for semi-high-throughput drug screening, with a potential of apical and basolateral dosing to mimic oral or systemic drug application. As shown in the results, the 3D liver tissue model elicits a stronger response to sub chronic drug exposure, mimicking the 9-week human trial, compared to monolayer and spheroids. Although we aimed to minimize donor-to-donor variability by using autologous hepatocytes across the monolayer, spheroid, and 3D organotypic liver tissue models, other factors may have contributed to the differences observed in the study. These factors include: (1) variations in culture protocols, (2) the presence of extracellular matrix proteins in the 3D organotypic model only, (3) specific culture conditions such as air–liquid interface (ALI) exposure in the 3D model, and (4) differences in media formulations and growth factor supplementation. Such elements may introduce biological variability, potentially accounting for the differential drug responses observed across the three liver models.

A key advantage of the platform used to generate the 3D organotypic liver tissue model is its ability to incorporate multiple cell types enabling the development of a more complex model as described previously (Ayehunie et al., 2018) for other *in vitro* tissue models. However, reliability and the predictive power of the newly developed 3D liver tissue model needs to be further validated in future studies using an expanded list of drugs selected from the FDA’s Liver Injury Rank (DILIrank) dataset. Additionally, incorporating other cell types such as Kupffer cells, stellate cells, and endothelial cells will enhance the model’s complexity and physiological relevance, thereby improving its potential to predict human responses to drug candidates.

Though regulatory pharmaceutical assessment currently relies on *in vivo* testing, significant differences exist between animal and human organ function in terms of drug permeation, distribution, metabolism, and clearance which can result in differences hepatotoxicity responses. In fact, animal models such as rats and dogs predict only about 50% of human drug-induced liver toxicity. Hence, extrapolation of animal-based hepatotoxicity results can lead to drug failure during human clinical trials [8] which is costly and results in loss of development time and resources. Anatomical differences between human and animal tissues suggest the need to develop well-structured human primary cell-based 3D hepatocyte tissue models to identify drug-induced hepatotoxicity, with an eventual goal of significantly reducing animals used to screen drugs for DILI in humans.

DILI is commonly caused by acute liver injury and is the leading cause of drug failure during clinical trials (Aithal et al., 2011; Weber and Gerbes, 2022). Several markers are currently used to characterize DILI, such as increased levels of the liver enzymes ALT and AST. Elevated levels of these markers are routinely used clinically as liver function tests, even though the release of the enzymes can also be associated with non-specific types of injury (Aithal et al., 2011; Katarey and Verma, 2016; Weber and Gerbes, 2022). For a tissue model to be a reliable metric for assessing risk of DILI, it must recapitulate the physiology and function of the *in vivo* counterpart. To this end, the reconstructed liver tissue expresses drug metabolizing enzymes and drug transporters, which are critical for accurately predicting drug-induced liver injury/toxicity.

Additionally, availability of the 3D reconstructed liver tissue model will provide significant benefits to the pharmaceutical industry by providing; 1) an apical and basolateral interfaces for testing drugs to mimic oral or intravenous drug administration, 2) a barrier tissue model that allows for quantitative TEER measurement for tissue barrier integrity, and 3) a high-throughput format that allows researchers to perform dose-response experiments with multiple test drugs to generate dose-response curves. Furthermore, the reconstructed tissue model allows the collection of apical washes and basolateral supernatants for the assessment of drug permeation, metabolism, and inflammatory markers, as well as cell viability assays. However, the current model has its own limitations and lacks the *in vivo* complexity of multiple cell types and vasculature. Additionally, the use of only N = 2 tissue replicates per drug exposure represents a limitation of this study. For future validation and standardization efforts, employing triplicate or more tissues per exposure and time point will be essential to ensure statistical rigor and to enhance the translational relevance of the 3D *in vitro* liver model.

In summary, the use of an *in vitro* liver tissue culture model during the early stages of drug development is critical to minimize hazard and potential health risk. Advantages of the *in vitro* model include: 1) cells are of human origin and thus are more physiological and predictive of human outcomes, 2) the drug metabolizing enzymes are stable and functional for weeks, 3) drug screening is more robust, cheaper, and faster, and 4) *in vitro* assays require smaller quantities of compounds compared to animal experimentation.

## 5 Conclusion

There is a great need for *in vitro* liver models that can be cultured for extended periods of time and exhibit organotypic morphology while maintaining a high level of major drug metabolizing enzyme expression. This model can serve as a platform to study acute and chronic liver toxicity. Histological and immunohistochemical analysis of this model showed a well polarized tissue structure capable of albumin production and expression of ZO-1 and CD-10. The tissue response to drug toxicity was stronger than that of the monolayer and spheroid models. The tissues successfully metabolized midazolam and drug induced liver injury was demonstrated following acute and chronic treatment with drugs known to cause liver toxicity in humans. Experimental results showed that increases in ALT and AST releases and a reduction in albumin production are reliable biomarkers to indicate potential liver damage. This study presents a novel *in vitro* human organotypic liver tissue model that is reproducible, easy to use, accurately predicts drug induced liver injury, and more closely resembles *in vivo* human tissue than spheroid models. The new 3D liver tissue model is anticipated to reduce the cost for compound screening, lower the risk of DILI in clinical trials, and decrease the scale of animal testing for drug-induced liver toxicity studies. However, the limited number of replicates used in this work may be a potential drawback, and the inclusion of additional tissue replicates per treatment is recommended for future studies.

## Data Availability

The original contributions presented in the study are included in the article/Supplementary Material, further inquiries can be directed to the corresponding author.
